# 

**Published:** 1859-06

**Authors:** 


### RECEIPTS FOR THE JOURNAL, UP TO JUNE 6, 1859.

### ILLINOIS.

| | | | |
|---|---|---|---|
| Dr. | G. W. Albin, | vol. 2, 1859, | $2.00 |
| " | Buckley, | " " | 2.00 |
| " | Ezekiel Friend, | " " | 2.00 |
| " | John Walker, | " " | 2.00 |
| " | R. F. Henny, | " " | 2.00 |
| " | J. P. Tucker, | " " | 2.00 |
| " | P. Myer, | " " | 2.00 |
| " | A. S. Haskell, | " " | 2.00 |
| " | L. A. Winslow, | " " | 2.00 |
| " | J. F. Marrh, | " " | 2.00 |
| " | A. J. McArthur, | " " | 2 00 |
| " | Leachman, | " " | 2.00 |
| " | Winston Somers, | " " | 2.00 |
| " | J C. Lowrie, | " " | 2.00 |
| " | Nance, | " " | 2 00 |
| " | C. M. Clark, | " " | 2 00 |
| " | David Prince, | " " | 2 00 |
| " | Wm. Buckworth, | " " | 2.00 |
| " | J. F. Weeks, | " " | 2.00 |
| " | R. S. Moloney, | vols. 1 & 2, 1858-9, | 4.00 |
| " | Bray, | vol. 1 & half of 2, " | 3.00 |
| Dr. | P. G. Corkins, | vols. 1 & 2, 1858-9, | $4.00 |
| " | A. Eldridge, | half v. 1 & v. 2, " | 3.00 |

### INDIANA.

| | | | |
|---|---|---|---|
| Dr. | W. Townsend, | vol. 2, 1859, | $2.00 |
| " | E H. Sutton, | " " | 2.00 |
| " | W. S. S. Cornett. | " | 1.00 |

### LOUISIANA.

| | | | |
|---|---|---|---|
| Dr. | G. J. Huey, | vol. 1, 1858, | $2.00 |

### WISCONSIN.

| | | | |
|---|---|---|---|
| Dr. | C. C. Warner, | vols. 1 & 2, 1858-9, | $4.00 |
| " | Henry A. Yeomans, | vol. 2, 1859, | 2.00 |
| " | James Pratt, | " " | 2.00 |

### IOWA.

| | | | |
|---|---|---|---|
| Dr. | E. Keith, | balance vol. 2, 1859, | $1.16 |

